# Multicenter Placebo-Controlled Randomized Study of Ethyl Pyruvate in Horses Following Surgical Treatment for ≥ 360° Large Colon Volvulus

**DOI:** 10.3389/fvets.2020.00204

**Published:** 2020-04-21

**Authors:** Lindsey M. Johnson, Susan J. Holcombe, Tara R. Shearer, Victoria Watson, Jeffery Gandy, Louise L. Southwood, Tymothy M. Lynch, Eric L. Schroeder, Callie A. Fogle, Lorraine M. Sordillo

**Affiliations:** ^1^Department of Large Animal Clinical Sciences, Michigan State University, East Lansing, MI, United States; ^2^Department of Pathobiology and Diagnostic Investigation, Michigan State University, East Lansing, MI, United States; ^3^Department of Clinical Studies, New Bolton Center, University of Pennsylvania, Kennett Square, PA, United States; ^4^Peterson and Smith Equine Hospital, Ocala, FL, United States; ^5^The Ohio State University, Department of Veterinary Clinical Sciences, Columbus, OH, United States; ^6^Department of Clinical Sciences, North Carolina State University, Raleigh, NC, United States

**Keywords:** colic, volvulus, horse, ethyl pyruvate, survival, large colon

## Abstract

Identifying therapies that mitigate ischemic colonic injury and improve mucosal healing and intestinal viability are crucial to improving survival in horses with ≥360° large colon volvulus (LCV). Ethyl pyruvate is the ethyl ester of pyruvate with diverse pharmacologic effects that limit ischemic injury and hasten intestinal mucosal repair in preclinical rodents, sheep and swine models. The objective of this study was to determine the effects of ethyl pyruvate on systemic indices of colon viability, expression of inflammatory genes in whole blood, morbidity and survival after surgical correction of LCV compared to controls. Horses received either 150 mg/kg ethyl pyruvate in 1 liter lactated Ringer's solution (LRS) or 1 liter LRS intravenously (IV) every 6 h for 24 h following surgical recovery for correction of LCV. Colic duration, perioperative heart rate (HR), packed cell volume (PCV), total solids (TS), blood L-lactate concentration, surgical time, intraoperative episodes of hypoxemia and hypotension, expression of inflammatory cytokine genes, fecal consistency and survival to hospital discharge were compared between ethyl pyruvate treated horses and controls. Twenty-two horses, 12 receiving ethyl pyruvate and 10 controls, were enrolled in the study. Ethyl pyruvate was safely administered to horses following surgical correction of LCV. No significant effects of ethyl pyruvate on post-operative variables, including survival, were found. Seven of 12 ethyl pyruvate treated horses and 5/10 controls survived to hospital discharge. Higher HR, PCV and blood L-lactate concentration at the time of hospital admission, *P* = 0.005, 0.01, 0.04, respectively, 24 h after surgery, *P* = 0.001, 0.03, 0.02, respectively, were associated with death. Heart rate, *P* = 0.005, 48 h after surgery was associated with death. Ethyl pyruvate was safely administered to horses following correction of LCV with no apparent adverse events but was not associated with improved post-operative outcomes including survival. A larger, randomized control trial is needed to fully evaluate the effectiveness of ethyl pyruvate. A major limitation of this investigation is the small sample size, making the study underpowered and creating a high possibility of type II error.

## Introduction

Large colon volvulus (LCV) is a painful and life threating form of colic that occurs when the colon rotates ≥360 degrees, resulting in colonic ischemia, colon and abdominal distension and cardiovascular compromise (1–6). Rapid surgical correction of the volvulus, with or without colon resection, and subsequent aggressive medical support are essential for survival. Even with prompt intervention, mortality ranges from 10–50% due to colonic devitalization (1–6). Survival is higher for horses near referral centers that receive timely surgical correction and approaches 90% (3). Prognosis is directly related to the viability and permeability of the large colon and ensuing development of systemic inflammatory response (1, 2). Goals of therapy in the immediate post-operative convalescent period include reducing systemic inflammatory response and normalizing cardiovascular function. A medication that mitigates intestinal injury and restores intestinal barrier function could improve survival, diminish morbidity and antimicrobial drug use, and hasten convalescence in horses with LCV.

Ethyl pyruvate is a stable lipophilic pyruvate derivative that effectively ameliorated structural and functional damage to the intestinal mucosa following mesenteric ischemia and hastened intestinal healing in preclinical studies of rodents (7, 8). Ethyl pyruvate has anti-inflammatory, anti-oxidant, and pro-metabolic activities facilitated, in part, by blocking elements of the NF-kB pathway, inhibiting apoptosis, and supporting cellular ATP synthesis (7, 9, 10). Treatment with ethyl pyruvate improved survival, cardiopulmonary parameters, and diminished intestinal injury in sheep (*n* = 14), mice (*n* = 51), rats (*n* = 24) and swine (*n* = 21), even when given up to 24 h after induction of ischemic injury, endotoxemia or sepsis (7–12). In a phase II clinical trial, ethyl pyruvate was administered to 51 human patients receiving cardiopulmonary bypass (13). The investigators determined that ethyl pyruvate conferred no benefit to these cardiac surgical patients but was safely administered to this high-risk group (13). A pilot study assessing the safety and efficacy of ethyl pyruvate was performed using 5 horses. A dose of 150 mg/kg delivered intravenously (IV) every 6 h was safely administered and diminished expression of proinflammatory genes in whole blood stimulated with endotoxin (14). In an *in vivo* equine endotoxemia model, ethyl pyruvate reduced pain scores and expression of proinflammatory genes compared to saline treated controls (15). Based on the promising results of ethyl pyruvate in preclinical models of ischemic intestinal injury and efficacy and safety data in horses, the objective of the current study was to evaluate the utility of ethyl pyruvate in horses with naturally occurring LCV. We hypothesized that ethyl pyruvate would improve survival and diminish morbidity and expression of proinflammatory genes in horses following surgical correction of LCV.

## Materials and Methods

### Study Design

This multicenter, randomized, placebo controlled clinical trial was conducted in accordance with the Animal Care and Use Committee at each participating institution and owner consent was obtained and CONSORT guidelines were implemented.

### Inclusion Criteria

Horses ≥1 year of age that underwent surgical correction of ≥360° LCV at one of the participating equine hospitals within the United States were recruited. If horses recovered from general anesthesia and were admitted to a hospital ward, they were eligible for inclusion in the study. Informed owner or agent consent was obtained prior to enrollment. Horse owners/agents were approached while the horse was in surgery once a diagnosis of LCV was made. In consultation with clinicians and surgeons horse owners/agents were informed of the LCV diagnosis, treatment options and recommendations. If the procedure continued owners were informed about the clinical trial and recruited to participate. No incentives were offered. A priori power analysis suggested that to demonstrate 50% improvement in survival 35 horses per treatment group were required.

### Anesthesia and Surgery

Acceptable surgical procedures included anatomic volvulus correction with or without pelvic flexure enterotomy, or large colon resection and side to side or functional end to end anastomosis. Horses with large colon resections were included because data suggests that survival following large colon resection vs. large colon volvulus correction and replacement were similar and rely on intestinal viability (3). If the colon was incised at surgery, a full-thickness, 1 cm by 2 cm sample of the pelvic flexure of the large colon was obtained and placed in 10% formalin and submitted to the Veterinary Diagnostic Laboratory at Michigan State University for preparation and analysis. No biopsy was taken if the colon was not incised. Episodes of hypoxemia (PaO2 < 60 mmHg) or hypotension (MAP < 60 mmHg) during anesthesia were obtained from the anesthesia record and surgery time was recorded (3).

### Treatments

**Figure 1:**
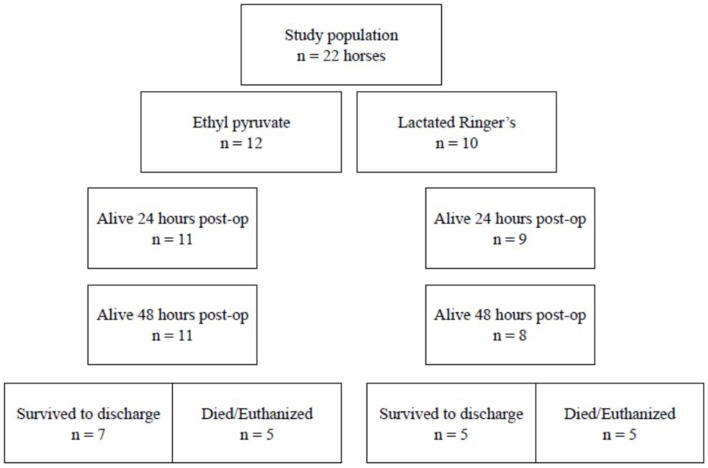
Flow diagram illustrating the grouping of horses in the study to assess the effect of ethyl pyruvate on horses following surgical correction of ≥ 360° large colon volvulus.

Following anesthetic recovery, horses were randomly allocated to receive the ethyl pyruvate^1^ treatment or the lactated Ringer's solution (LRS) control, Figure 1. Randomization was attempted by selecting a number from an envelope. Each of the five participating hospital received an envelope containing 10 consecutive numbers. Even numbers received ethyl pyruvate treatment and odd numbers received LRS control. The horse's study number was used to label the PAXgene^2^ tubes and colon biopsy to maintain anonymity and blinding for histopathology and gene expression analysis. The horse's study number was also placed on the data collection sheet, Supplement 1, assigned to each enrolled horse. Signalment, owner information, perioperative variables and survival information were recorded on the data collection sheet. The first treatment or control was administered after the horse was admitted to its stall following anesthetic recovery. Horses received either 150 mg/kg ethyl pyruvate in 1 liter LRS, as an intravenous continuous rate infusion (CRI) over 60 min or 1 liter LRS as a CRI over 60 min. Continuous rate infusion rather than bolus infusion was performed because the only reported complication of ethyl pyruvate administration was excitement if ethyl pyruvate was administered too quickly in people and mice (personal communication, Mitchell Fink). The ethyl pyruvate solution reached room temperature, was withdrawn from the bottle and added to 1 liter of LRS using a 0.22 μm sterile millipore filter^3^. The treatment and control infusions were administered every 6 h for a total of 4 infusions. Additional monitoring and treatments including intravenous fluids, antimicrobials, non-steroidal anti-inflammatories and analgesics, and other adjunctive therapies were prescribed by the attending veterinarian/s.

### History and Clinical Variables

Duration of colic pain, pregnancy and foaling status were provided by horse owners or agents. The number of hours between the horse first showing signs of acute abdominal pain and the beginning of surgery established the duration of colic pain. Body weight was determined prior to surgery or in severely painful horses following anesthetic recovery by weighing the horse on a scale. Heart rate was determined at the time of hospital admission, 24 and 48 h after surgery. Additional patient monitoring was prescribed by the attending veterinarian/s. Quality of feces passed, including no feces, normal feces, diarrhea, or bloody diarrhea was recorded at 24 and 48 h after surgery. Number of days the horse received antimicrobial drugs, the number of hospitalization days, and short-term outcome including lived to hospital discharge, died or was euthanized in the hospital were recorded. Cause of death or reason for euthanasia were confirmed by post mortem examination or explained by the attending clinician. Owners/trainers were contacted by telephone to determine if horses were alive, performance status, or reasons for death/euthanasia.

### Blood Sample Collection

Horses had blood sampled and PCV, TS, and L-lactate^4^ concentrations determined at the time of hospital admission, 24 and 48 h after surgery. Each horse had whole blood collected immediately prior to the first treatment and within 30 min following the last treatment in PAXgene tubes designed specifically for the collection and stabilization of cellular RNA from whole blood. The blood was obtained by venipuncture following aseptic preparation from the jugular vein or aseptically from the intravenous catheter. PAXgene tubes were inverted 8–10 times and stored at room temperature for 24 h per manufacturer instructions. Following collection of the second PAXgene tube, both PAXgene tubes and the intestinal biopsy (if taken) were shipped overnight to the Michigan State University Veterinary Diagnostic Laboratory. Biopsies were submitted to the histopathology laboratory for preparation and PAXgene tubes were frozen at −20°C for batch analysis of inflammatory cytokine gene expression.

### RNA Isolation From Whole Blood

Blood was collected before and after ethyl pyruvate treatment or LRS control to measure the expression of inflammatory genes including tumor necrosis factor alpha (TNF-α), IL-1, IL-6 and high mobility group box-1 (HMGB1). Using the PAXgene Blood RNA Kit^5^, whole-blood RNA was extracted, according to manufacturer's instruction (Qiagen). DNAse I was used to prevent genomic DNA contamination. The extracted total RNA was analyzed for purity using a nanodrop and bioanalyzer. Cleanup and purification of the total RNA extracted was accomplished with the RNase-Free DNase Set^6^. Following cleanup, all samples had an optical density between 1.9 and 2.2. Until samples were processed for cDNA synthesis they were stored at −80°C.

### Real-Time-PCR

Purified RNA was converted to cDNA using a High-Capacity cDNA Archive Kit^7^. Real-time relative quantification PCR was performed with a 7,500 Fast Real-Time PCR system^8^ using pre-designed TaqMan MGB probes^9^ from Applied Biosystems. PCRs were performed in triplicate using a 20 ul reaction mixture per well, containing 10 ul of TaqMan Gene Expression Master Mix (2X) (Applied Biosystems) 1 ul of (20X) Custom TaqMan® Gene Expression Assay Mix (Applied Biosystems), 5 ul of amplified cDNA, and the balance was Nuclease-free water. A (20X) pre-designed Taqman® Gene Expression Assay for beta actin, GUSB, and B2M from Applied Biosystems was used as an endogenous control. Gene expression was calculated using the ΔCt method for statistical analysis. All equine Taqman assays are displayed in Table 1.

**Table 1:** Gene targets and assay numbers for qPCR.

**Taqman assay**	**Assay number**
HMGB1	Ec03469480_g1
IL-10	Ec03468470_m1
IL-6	Ec03468678_m1
TNFα	Ec03467871_m1
Beta actin	Ec04176172_gh
B2M	Ec03468700_m1
GUSB	Ec03470630_m1

*HMGB1—high mobility group box 1; IL—interleukin; TNFα—tumor necrosis factor alpha; B2M—beta 2 microglobulin; GUSB—glucuronidase beta*.

### Histopathology

Colon biopsies were routinely processed, paraffin embedded, sectioned at a thickness of 5 μm, and stained with hematoxylin and eosin at the Michigan State University Veterinary Diagnostic Laboratory. Microscopic examination of each tissue was performed by a boarded veterinary anatomic pathologist (VW) that was blinded to identity, treatment group, and outcome of the horse. The severity of epithelial loss and mucosal hemorrhage was graded using a previously published scoring system (2). The number of mucosal eosinophils was determined for each section by counting the eosinophils present in a 0.25 × 0.25 mm area using a 1 cm^2^ 10 × 10 grid reticle at 400X within 3 randomly selected microscopic fields.

### Data Analysis

Variables from the horses' data collection sheets were collated. Continuous data, including age, colic duration, heart rate, PCV, TS, L-lactate concentration, surgery time, body weight, interstitium to crypt ratio (I:C) ratio, crypt length, and number of eosinophils and gene expression using ΔCt were reported as mean ± standard deviation if the data were normally distributed or as median (50% interquartile range {Q1–Q3}) if the data were not normally distributed. Normality was assessed using the Shapiro-Wilk test, histogram analyses and normal probability plots. Episodes of hypoxemia (PaO_2_ < 60 mmHg) and hypotension (MAP < 60 mmHg), fecal consistency scores, sex, enterotomy, colon resection, and pregnancy were compared between the treatment and control groups and between horses that lived vs. horses that died or were euthanized using the Fisher's exact test. Histologic hemorrhage scores were compared using Mann-Whitney U test. A split plot repeated measures ANOVA with three grouping factors including treatment, surgical hospital and survival, and one repeat factor, time, was performed. Normality of the residuals was assessed by plotting histograms and determining that the distribution was unimodal and symmetrical, showing a normal probability plot and using the Shapiro-Wilk test. Grouping factors were removed and the split-plot ANOVA repeated until significance was determined. The final model contained one grouping factor, survival, and one repeat factor, time. *Post-hoc* power analysis for treatment was performed. Statistical analyses were performed using SAS^10^ and *P* < 0.05 was considered significant.

## Results

### Horses

Twenty-two horses were included in the study from 5 participating hospitals from September 2017–December 2018. Hospital A enrolled 4 horses, hospital B enrolled 2 horses, hospital C enrolled 5 horses, hospital D enrolled 2 horses, and hospital E enrolled 6 horses. Twelve horses received the ethyl pyruvate treatment and 10 horses received the LRS control, Figure 1. Breeds represented in the ethyl pyruvate treatment group included Thoroughbreds = 6, Quarter Horses = 3, Saddlebred = 1, Friesian = 1, Palomino = 1. Control horses included Thoroughbreds = 4, Warmbloods = 2, Quarter Horse = 1, Percheron = 1, Morgan = 1, and 1 Standardbred. The ethyl pyruvate treatment group contained 4 geldings, 7 mares, and 1 stallion compared to the control group with 6 geldings and 4 mares. The mean ± standard deviation age of the ethyl pyruvate treatment horses was 10.1 ± 6.3 years compared to 13.2 ± 4.76 years for the control horses, *P* = 0.2. Three of the 10 controls were confirmed pregnant at the time of surgery and none of the ethyl pyruvate treatment horses were pregnant.

### Ethyl Pyruvate Treatment vs. LRS Controls

Treatment was removed from the split-plot ANOVA model because treatment had no significant affect (*P* > 0.30) on any of the variables measured including heart rate, PCV, TS, L-lactate concentration, inflammatory gene expression, hospitalization day or survival, Table 2. Five of 10 controls and 7 of 12 ethyl pyruvate treated horses survived. To detect an 8% difference between the treatment and control groups if true mortality in untreated controls was 50% would have required 391 horses per group.^10^

**Table 2:** Peri-operative variables measured in 12 horses that received 150 mg/kg ethyl pyruvate treatment and 10 control horses that received lactated Ringer's solution following surgical correction of ≥360 degree large colon volvulus.

**Variable**	**Treatment**	**Time**	***N* =**	**Mean**	**SD**	**95% CI**	**Median**	**Minimum**	**Maximum**	***P* =**
Body weight kg	Ethyl pyruvate	Ad	12	521	77	450, 575	527	397	672	0.3
	Control		10	578	144	501, 557	541	485	954	
Colic duration hrs.	Ethyl pyruvate	Ad	12	7	7	3, 9	4	3	24	0.7
	Control		10	10	14	3, 7	6	1	48	
Surgery time min.	Ethyl pyruvate		12	126	46	92, 171	113	55	190	0.4
	Control		10	146	64	105, 167	125	75	300	
Days in hospital	Ethyl pyruvate		12	6	2	4, 8	6	1	9	0.6
	Control		10	8	6	4, 12	7	1	20	
Heart rate bpm	Ethyl pyruvate	Ad	12	72	28	54, 89	70	36	120	0.4
		24	11	55	17	44, 66	48	40	84	0.5
		48	11	52	17	41, 63	44	36	80	0.9
	Control	Ad	10	64	28	44, 84	58	32	120	
		24	9	60	16	48, 73	56	42	84	
		48	8	50	15	37, 62	46	36	80	
PCV %	Ethyl pyruvate	Ad	12	46	10	39, 52	46	30	70	0.7
		24	11	37	9	32, 43	36	21	50	0.8
		48	11	38	9	32, 43	35	22	53	0.8
	Control	Ad	10	47	6	43, 51	50	35	53	
		24	9	38	6	34, 43	36	30	50	
		48	8	35	7	29, 41	35	25	50	
Total solids g/dL	Ethyl pyruvate	Ad	12	6.9	1	6.2, 7.6	7.1	5.2	9	0.5
		24	11	5.9	0.8	5.4, 6.4	5.8	4.8	7.6	0.7
		48	11	6.1	0.9	5.5, 6.7	6.4	5	7.8	>0.9
	Control	Ad	10	7.2	0.9	6.6, 7.8	6.8	6.2	8.8	
		24	9	5.6	0.7	5.1, 6.1	5.5	5	6.6	
		48	8	6.1	0.9	5.3, 6.8	6.2	5	7.2	
L-lactate mmol/L	Ethyl pyruvate	Ad	12	5.7	3.6	3.5, 8.0	5.05	1.2	13	0.3
		24	11	1.3	0.7	0.8, 1.7	1.1	0.5	2.9	0.6
		48	11	1	0.4	0.7, 1.3	0.9	0.5	1.8	0.6
	Control	Ad	10	4.7	3.2	2.4, 7.0	4.25	1.3	12.2	
		24	9	1.8	1.9	0.3, 3.3	1.2	0.2	6.3	
		48	8	1.6	0.8	1.0, 2.3	1.25	0.7	2.8	

*The results of a split-plot ANOVA with one grouping factor, treatment, and one repeat factor, time, demonstrating that perioperative variables measured in ethyl pyruvate treatment horses that received 150 mg/kg ethyl pyruvate in 1 liter lactated Ringer's solution (LRS), intravenously (IV) as a continuous rate infusion (CRI) every 6 h for 4 treatments following surgical recovery for large colon volvulus were not significantly different compared to the perioperative variables measured in control horses that received 1 liter LRS, IV, as a CRI every 6 h for 4 treatments following surgical recovery for large colon volvulus. SD = standard deviation; CI = confidence interval; kg = kilograms; hrs = hours; min. = minutes; bpm = beats per minute; % = percent; g/dL = grams per deciliter; mmol/L = millimoles per liter*.

No significant differences were determined for admissions variables between the ethyl pyruvate treatment and control horses. Ethyl pyruvate treatment horses had higher admissions heart rate and L-lactate concentrations compared to the control horses which may have impacted survival and create bias favoring survival of the controls compared to the ethyl pyruvate treated horses. Mean body weight was higher and mean surgery time and median colic duration were longer for controls compared to treatment horses which also may have created bias, favoring improved survival in the treatment group. Eight of 10 control horses and 8 of 12 ethyl pyruvate treated horses had pelvic flexure enterotomies performed, *P* = 0.7. One control horse and 1 ethyl pyruvate treated horse had large colon resection performed, *P* > 0.9. Four of 10 control horses and 6 of 12 ethyl pyruvate treated horses developed hypotension, PaO_2_ < 60 mmHg, *P* = 0.7. Two of 10 controls and 2 of 12 ethyl pyruvate treated horses developed hypoxemia, PaO_2_ < 60 mmHg, *P* > 0.9. Horses tolerated the ethyl pyruvate treatment and control infusions well and no adverse events were reported during the 60-min ethyl pyruvate or the 60-min LRS control infusion.

Twenty-four hour post-operative data were obtained for 11 of 12 ethyl pyruvate treated horses and 9 of 10 control horses. One ethyl pyruvate treated horse and one control horse died in their stalls prior to the 24-h post-operative timepoint. An additional control horse was euthanized at 36 h after surgery, such that 11 ethyl pyruvate treated horses and 8 LRS control horses remained in the study at 48 h after surgery. Only quality of the feces passed at 48 h after surgery differed significantly between the ethyl pyruvate treatment and control horses. Equine hospital was also removed from the split-plot ANOVA model because hospital had no significant effect (*P* > 0.50) on any of the variables measured.

Five of 10 LRS treated control horses died or were euthanized. Post-mortem diagnoses included multiple infarcts of the large colon in one horse, devitalized large colon with septic peritonitis in 2 horses, and 3 horses had devitalized large colon reported. Five of 12 ethyl pyruvate treated horses died or were euthanized. Two ethyl pyruvate treated horse had devitalized large colon at post-mortem examination. A third ethyl pyruvate treated horse suffered a second large colon volvulus 7 days after surgery confirmed at post mortem and was euthanized. This mare was receiving no medications or supplemental fluids and eating a full ration of hay. A fourth ethyl pyruvate treated horse was euthanized following diagnosis of a chronic mandibular fracture that occurred prior to development of colic. No post mortem was performed on this individual. A fifth ethyl pyruvate treated horse was euthanized due to progressive azotemia and financial constraints 2 days after surgery but was stable, eating hay and passing normal manure. No post mortem was performed on this horse.

Histomorphology of the colon *- Nine* of the twenty-two horses enrolled in this study had colonic pelvic flexure biopsies obtained at surgery. Six of the horses were treated with ethyl pyruvate and three were control horses. No significant differences in the scores or histomorphologic measurements between the ethyl pyruvate treatment horses and controls were found, Table 3.

**Table 3:** Results of pelvic flexure biopsies taken from 9 of 22 enrolled horses at the time of surgical correction of LCV.

**Horse**	**Treatment**	**Hemorrhage scores**	**Epithelial loss**	**I:C**	**Crypt length**	**Eos. #**	**Survived**
1	EP	4	4	1.6 ± 0.3	214 ± 43	2	Yes
10	EP	3	1	1.0 ± 0.5	252 ± 26	43	Yes
15	EP	3	3	1.3 ± 0.16	258 ± 28	43	No
19	EP	3	1	1.5 ± 0.8	246 ± 15	58	Yes
31	EP	3	3	1.3 ± 0.4	297 ± 43	47	Yes
56	EP	0	1	1.5 ± 0.5	437 ± 103	75	Yes
	EP mean	2.7 ± 1.4	2.2 ± 1.3	1.4 ± 0.4	284 ± 43	44.7 ± 24	
20	Control	3	2	0.5 ± 0.1	417 ± 47	50	Yes
59	Control	0	2	1.4 ± 0.2	352 ± 68	48	No
33	Control	4	2	1.75 ± 0.8	370 ± 188	33	No
	Control mean	2.3 ± 2.1	2 ± 0	1.2 ± 0.6	379 ± 101	43.7 ± 9	
	*P* value Treatment	>0.9	>0.9	>0.9	0.2	>0.9	
	P value Survival	>0.9	0.6	0.6	0.7	0.4	

*The estimated percentage of epithelial loss affecting the surface of the mucosa was graded from 1 to 4 where grade 1 = 0–25%; 2 = 25–50%; 3 = 50–75%; and 4 = 75–100% (2). The degree of mucosal hemorrhage was classified based on a scale from 0 to 4 where 0 = no hemorrhage; 1 = few individual red blood cells within the lamina propria; 2 = increased number of individual red blood cells within the lamina propria; 3 = aggregates of red blood cells within the lamina propria; and 4 = confluent red blood cells obscuring the lamina propria (2). In addition to subjective measurements, the interstitium to crypt ratio (I:C ratio), crypt length, and number of eosinophils were determined. The interstitium to crypt ratio, or the ratio of measured lamina propria space occupied by the interstitium and the space occupied by the crypts, and the crypt length were measured in 3 randomly selected microscopic fields. The number of mucosal eosinophils was determined for each section by counting the eosinophils present in a 0.25 × 0.25 mm area using a 1 cm^2^ 10 × 10 grid reticle at 400X within 3 randomly selected microscopic fields. EP - ethyl pyruvate; I:C—interstitial to crypt ratio; Eos.# - number of eosinophils in a 0.25 × 0.25 mm area using a 1 cm^2^ 10 × 10 grid reticle at 400X within 3 randomly selected microscopic fields*.

**Figure 2:**
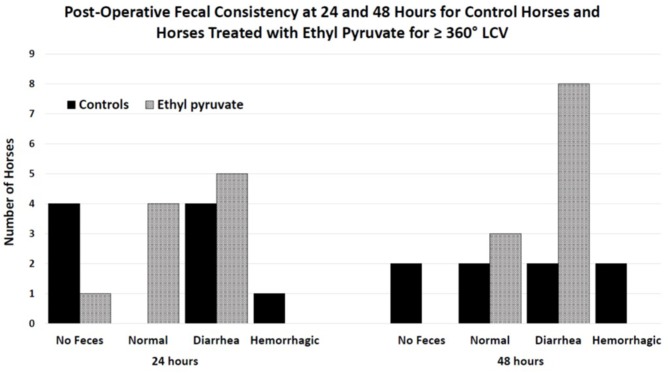
Histogram of fecal consistency for horses 24 and 48 h after surgical correction of large colon volvulus. Fecal consistency was described as no fecal production, producing normal feces, diarrhea or hemorrhagic diarrhea. Diarrhea was defined as unformed feces. No significant differences were found when comparing controls treated with 1-liter lactated Ringer's solution (LRS) every 6 h for 4 treatments with horses treated with 150 mg/kg ethyl pyruvate in 1-liter LRS every 6 h for 4 treatments post operatively following anesthetic recovery when the horse returned to its stall. Quality of feces was likely impacted by performing a pelvic flexure enterotomy, timing of feed, and quality of feed.

Variables associated with death – Only the grouping factor survival and time remained in the split-plot ANOVA model. Overall survival was 56% (12/22), with 50% of control horses (5/10) and 58% (7/12) of ethyl pyruvate horses surviving to be discharged from the hospital. Admission variables significantly associated with death included days in hospital, heart rate, PCV, and L-lactate concentrations, Table 4. Intraoperative variables associated with death included episodes of hypoxemia (PaO2 < 60 mmHg), *P* = 0.03, and hypotension (MAP < 60 mmHg), *P* = 0.008 under general anesthesia. Variables measured 24 h after surgery that were significantly associated with death included heart rate, PCV, and blood L-lactate concentrations. At 48 h after surgery, heart rate was significantly associated with death. Expression of inflammatory genes, including TNFα, IL-6, IL-10, and HMGB-1 following anesthetic recovery prior to treatment and 24 h after surgery were not significantly different between horses that survived and horses that did not survive, Table 5. Colic duration prior to surgery, surgery time and body weight were not significantly different between horses that survived compared to horses that died or were euthanized. There was no significant difference between horses that lived or did not survive, for sex, *P* = 0.4, or pregnancy status, *P* = 0.4, enterotomy or colon resection, *P* > 0.9 for both. There was no significant difference between survivors and horses that did not survive for the quality of feces passed at 24 or 48 h after surgery, *P* = 0.9 and 0.9, respectively, Figure 2.

**Table 4:** Perioperative variables measured in horses that lived vs. horses that died following surgical correction of ≥ 360 degree large colon volvulus.

**Variable**	**Group**	***N***	**Mean**	**SD**	**95% CI**	**Median**	**Minimum**	**Maximum**	***P***
Body weight (kg)	Lived	12	520	70	478, 550	523	397	672	0.3
	Died	10	580	148	485, 585	545	440	954	
Colic duration hrs	Lived	12	8.3	6.9	3.5, 11.5	6	1	24	0.3
	Died	10	8.4	14	3, 6	4	2	48	
Ad Heart rate bpm	Lived	12	50	14	40, 58	50	32	80	0.005
	Died	10	89	25	80, 110	90	44	120	
24 Heart rate bpm	Lived	12	47	9	41, 48	44	40	74	<0.001
	Died	8	73	10	67, 82	74	56	84	
48 Heart rate bpm	Lived	12	43	10	36, 44	42	36	72	0.005
	Died	7	65	14	52, 80	60	48	80	
Ad PCV %	Lived	12	42	7	36, 47	44	30	53	0.01
	Died	10	51	7	49, 52	50	44	70	
24 PCV %	Lived	12	34	5.8	33, 36	35	21	43	0.03
	Died	8	43	6.6	40, 48	44	30	50	
48 PCV %	Lived	12	33	4.3	32, 36	35	22	38	0.07
	Died	7	42	10	34, 50	46	25	53	
Ad TS g/dL	Lived	12	7	0.8	6.3, 7.4	7	6.2	8.4	0.8
	Died	10	7.1	1.2	6.6, 7.6	7	5.2	9	
24 TS g/dl	Lived	12	5.9	0.8	5.2, 6.3	5.9	5	7.6	0.3
	Died	8	5.6	0.7	5, 6.1	5.4	4.8	6.6	
48 TS g/dL	Lived	12	6.3	0.8	5.7, 6.8	6.5	5	7.8	0.1
	Died	7	5.7	0.8	5, 6.4	5.2	5	7.2	
Ad L-lactate mmol/L	Lived	12	4.1	3.3	2, 5	3.1	1.2	13	0.04
	Died	10	6.6	3	5.1, 7.5	6.4	2.3	12.2	
24 L-lactate mmol/L	Lived	12	1	0.7	0.6, 1.3	0.8	0.5	1.7	0.02
	Died	8	2.2	1.8	1.2, 2.6	1.5	1.2	6.3	
48 L-lactate mmol/L	Lived	12	1.1	0.6	0.8, 1.3	0.9	0.7	2.4	0.07
	Died	7	1.6	0.7	1.1, 2.3	1.2	1	2.8	
Surgery time min	Lived	12	118	39	93, 145	113	55	180	0.22
	Died	10	155	65	100, 180	163	75	300	
Hospital days	Lived	12	8	4	6, 10	7	4	20	0.03
	Died	10	5	4	1, 7	4	1	12	

*The results of a split-plot ANOVA with one remaining grouping factor, survival, and one repeat factor, time. Neither treatment, ethyl pyruvate vs. lactate Ringer's solution control, or hospital were significant and were removed from the model. Bolded values were significantly different between horses that lived and horses that died following surgical correction of large colon volvulus, P < 0.05. SD = standard deviation; CI = confidence interval; kg = kilograms; hrs = hours; bpm = beats per minute; % = percent; g/dL = grams per deciliter; mmol/L = millimoles per liter; min = minutes*.

**Table 5:** Inflammatory gene expression in whole blood for horses following surgical correction of ≥ 360 degree large colon volvulus that lived and horses that died before and after treatment with 150 mg/kg ethyl pyruvate or lactated Ringer's solution control.

**Gene**	**Treatment**	**Survive**	**Time**	**N =**	**Mean**	**Std Dev**
IL-10	Control	Lived	PO	5	6.6	1.2
			24 PO	5	9.4	1.3
		Died	PO	5	6.4	2.1
			24 PO	5	8	1.8
	Ethyl pyruvate	Lived	PO	7	6.7	2.1
			24 PO	7	8.6	1
		Died	PO	5	7.1	0.9
			24 PO	5	7.5	0.2
HMGB-1	Control	Lived	PO	5	3	0.6
			24 PO	5	2.8	0.5
		Died	PO	5	3.1	0.1
			24 PO	5	2.3	0.4
	Ethyl pyruvate	Lived	PO	7	2.3	1
			24 PO	7	2.4	0.8
		Died	PO	5	3.2	2.3
			24 PO	5	3.5	1.3
IL-6	Control	Lived	PO	5	10.2	2
			24 PO	5	13.2	2
		Died	PO	5	13.1	2.2
			24 PO	5	12.1	1.3
	Ethyl pyruvate	Lived	PO	7	11.8	1.3
			24 PO	7	12.3	1.4
		Died	PO	5	10	6.2
			24 PO	5	12.6	0.7
TNFa	Control	Lived	PO	5	5	0.7
			24 PO	5	3.9	1.1
		Died	PO	5	5.4	1.9
			24 PO	5	4.7	1.1
	Ethyl pyruvate	Lived	PO	7	5.3	0.5
			24 PO	7	5.1	0.6
		Died	PO	5	6	0.1
			24 PO	5	6	0.1

*Inflammatory gene expression as ΔCT in whole blood from horses after surgical correction of large colon volvulus. Blood samples were collected post-operatively (PO) when horses returned to their stalls following anesthetic recovery and 24 h later (24 PO) after treated horses received 150 mg/kg ethyl pyruvate in 1 liter lactate Ringer's solution (LRS), intravenously (IV) as a controlled rate infusion (CRI) every 6 h for 4 treatments or control horses received 1 liter LRS, IV as a CRI, every 6 h for 4 treatments*.

Six of the 7 horses treated with ethyl pyruvate survived as of November 2019, a mean ± standard deviation 634 ± 189 days. Two horses are competitive event horses, one horse is racing and is a grade 1 stakes winner, one horse is racing successfully, one horse is a gaited show horse, and 2 horses are retired. One ethyl pyruvate treated horse colicked 1 year after surgery and was euthanized without surgery. All five of the control horses that were discharged from the hospital were alive as of November 2019 and survived 564 ± 87 days. Three are show horses, one is a brood mare in foal, and one horse is used for trail riding.

## Discussion

We showed, for first time, that ethyl pyruvate was successfully administered and caused no apparent complications in clinical equine patients following surgical correction of naturally occurring LCV. We were unable to show any significant effect of ethyl pyruvate treatment on any of the clinical variables measured, including post-operative heart rate, PCV, L-lactate concentration and survival, refuting the study hypothesis. Due to low case enrollment numbers, the clinical trial was under-powered, limiting the impact of the results.

The dose, 150 mg/kg, and dosing interval, every 6 h, of ethyl pyruvate used in this study were established based on previous work in horses but may have been insufficient to demonstrate clinical efficacy in this study (14, 15). Ethyl pyruvate doses in animal studies ranged from 40–150 mg/kg and people received 100 mg/kg during a phase II clinical trial (9–15). Mice were treated with 1,000 mg/kg ethyl pyruvate intraperitoneally following inoculation with *Salmonella typhimurium* and showed improved intestinal histomorphology and barrier function as compared to controls (16). Future work in horses may establish alternate doses, dosing intervals, and alternate routes, including intraperitoneal administration at surgery.

Failure to detect significant improvement in survival in the ethyl pyruvate treated horses may have occurred for several reasons including lack of statistical power, the variability associated with a naturally occurring disease population, insufficient ethyl pyruvate dosing, or a true ineffectiveness of ethyl pyruvate to diminish hemorrhagic intestinal injury in horses. In the current study, survival for control horses was 50% compared to 58% for horses receiving ethyl pyruvate. *Post-hoc* power analysis revealed that 391 horses per group were required to detect significant effects of treatment in this study, enrollment far beyond the reach of this study^10^. Survival following LCV is reportedly 43–88% (1–6). Survival is dependent on colic duration prior to surgical correction which correlates closely with ischemic intestinal injury (1–3). Work by Hackett and colleagues showed that 92% of horses that had surgery within 2 h of showing colic signs survived (3). Horses were 3 times and 12 times more likely to die when the duration of colic prior to surgery was 2–4 h or > 4 h, respectively (3). Duration of colic for horses in the current study was a median of 6 h ranging from 1–24 h. The wide range of colic duration and associated intestinal ischemia within the study population likely contributed to the variability of disease severity. Death following LCV is usually associated with devitalized colon and associated cardiovascular instability but may occur for other reasons (3). Devitalization of the large colon occurred in 5 control and 2 ethyl pyruvate treated horses. Three horses that received ethyl pyruvate were euthanized for reasons (mandibular fracture, azotemia/finances, repeat LCV) perhaps ancillary to large colon viability and may have impacted the interpretation of the survival results.

The actual time between volvulus occurrence, intestinal injury, and ethyl pyruvate treatment is unknown. Given that the median duration of colic prior to hospital admission was 6 h and mean surgery time was approximately 2 h, it is reasonable to estimate that the time between intestinal injury and treatment was 10–12 h. Ethyl pyruvate showed beneficial effects in animals even when administered 12–24 h after intestinal injury or sepsis induction (7, 9, 10, 12, 16–18). However, earlier administration of ethyl pyruvate, for example during surgery, may be required to demonstrate efficacy in horses with intestinal injury. Supporting cardiopulmonary function in horses anesthetized for correction of LCV may improve survival given that episodes of hypoxemia and hypotension were significantly higher in horses that did not survive compared to survivors in the current study and previous reports (3). These hemodynamic events may cause low flow colon ischemia, exacerbating the injury caused by the volvulus. Ethyl pyruvate significantly improved hemodynamic parameters, including cardiac output and oxygen delivery, in anesthetized research horses and had no detrimental effects on anesthetic recovery [Munoz, K. unpublished data] suggesting that intraoperative administration of ethyl pyruvate could be pursued in horses with colic.

A common sequela of large colon volvulus was abnormal manure production (3, 19–21) likely due to colonic mucosal damage and loss of surface area for fluid absorption (19). Abnormal manure consistency was associated with death in horses following surgical correction of LCV (3). Sixty-four percent of non-surviving horses had abnormal post-operative manure consistency ranging from loose to hemorrhagic diarrhea, whereas only 4.5% of non-surviving horses in the Hackett study passed normal manure (3). The differences in the quality of feces passed post-operatively between the ethyl pyruvate treated and control horses in the current study may have been related to protective effects of ethyl pyruvate on large colon intestinal integrity. Horses treated with ethyl pyruvate did not develop hemorrhagic diarrhea and were all passing feces 48 h after surgery compared to untreated controls that did develop hemorrhagic diarrhea or had yet to produce manure at 48 h post-surgery. However, Hackett et al. reported that abnormal manure production, including diarrhea and hemorrhagic diarrhea were both associated with non-survival (3) making interpretation of the fecal consistency results in the current study unclear.

No significant differences were detected for gene expression of HMGB1, TNFα, IL-6 and IL-10 in whole blood for horses treated with ethyl pyruvate compared to control horses or between horses that lived compared to horses that did not survive.

Previous work demonstrated that ethyl pyruvate decreased whole blood gene expression of TNFα and IL-6 in horses pretreated with endotoxin compared with control horses and diminished proinflammatory gene expression in equine mononuclear cells and whole blood treated with lipopolysaccharide (14, 15, 22). In the *in vivo* equine endotoxemia study, proinflammatory gene expression was measured in blood samples that were collected 60–300 min following endotoxin infusion in research horses. Expression of both TNFα and IL-6 peaked at 60 and 120 min after endotoxin administration. Blood samples for inflammatory gene expression were obtained following anesthetic recovery and 24 h after surgery in the current clinical trial, possibly missing the relevant time points for peak expression of HMGB1, TNFα, IL-6, and IL-10. Inability to detect differences in gene expression between the ethyl pyruvate treated and control horses or horses that lived vs. horses that did not survive in the current study may be due to the timing of the measurements of inflammatory gene expression, effects of other medications that the horses received including lidocaine and non-steroidal anti-inflammatory drugs, and the development of endotoxin tolerance by the horses (23).

Like previous reports, horse survival was associated with more days of hospitalization (3) lower perioperative heart rate (1), PCV and blood L-lactate concentrations (1, 6). Increased PCV and heart rate are indicative of hemodynamic events associated with endotoxemia and sepsis resulting from colonic ischemia and death (3). Gonzalez and colleagues reported that histologic colon hemorrhage scores ≥3, suggestive of more severe hemorrhagic ischemic injury, predicted death in horses with LCV. In their study, 7 of 13 (54%) horses with hemorrhage scores ≥3 did not survive and 30 of 34 (88%) horses with hemorrhage scores <3 survived (2). In the current study hemorrhage scores were not associated with survival; however, the number of biopsies in this study was lower compared to Gonzalez et al. One of 5 (20%) horses treated with ethyl pyruvate and 1 of 2 (50%) of control horses with hemorrhage scores ≥3 died or were euthanized in hospital. Four of five ethyl pyruvate treated horse with hemorrhage scores ≥3 survived, suggesting that ethyl pyruvate treatment may benefit colon healing. We were unable to obtain colonic biopsies following administration of ethyl pyruvate to determine if ethyl pyruvate improved biopsy scores due to the clinical nature of the study.

The biggest limitation of this study was the small sample size, creating a high possibility of a type II error, thereby restricting our ability to detect significant effects of ethyl pyruvate in horses with LCV. The severity of LCV disease was impacted by duration of colic symptoms, degree of cardiovascular compromise, and the extent of colonic incarceration, which may have biased comparisons of ethyl pyruvate treated cases to LRS-treated controls. Post-operative treatment was prescribed by attending clinicians and was not standardized. Therapies were variable and included intravenous crystalloid fluids, lidocaine continuous rate infusions, antimicrobial therapy, non-steroidal anti-inflammatory medications and other adjunctive treatments. Timing, quantity and quality of feed offered to enrolled horses was not standardized and may have impacted the results of the study.

Motivation to explore the clinical efficacy of ethyl pyruvate in LCV was based on the lack of currently available treatments to improve intestinal viability and horse survival. The results of this study did not support the hypothesis that ethyl pyruvate would significantly improve survival in horses following surgical correction of LCV. Results in the present study provide important safety and dosing information for future studies. A larger, randomized control clinical trial is required to assess the value of ethyl pyruvate in horses with LCV. Safe administration of ethyl pyruvate in the post-operative period to horses and the utility of ethyl pyruvate treatment in animals with intestinal ischemia (7, 8), infectious intestinal disease (16), and adhesion formation (24, 25) suggest that ethyl pyruvate may have applications in horses with other forms of intestinal disease. Unfortunately, the impact of the current study results were limited by the low study power.

## Data Availability Statement

Data available upon request from the corresponding author.

## Ethics Statement

The animal study was reviewed and approved by Animal Use and Care Committee, Michigan State University; Animal Use and Care Committee, The Ohio State University; Animal Use and Care Committee, North Caroline State University; Animal Use and Care Committee, University of Pennsylvania. Written informed consent was obtained from the owners for the participation of their animals in this study.

## Author Contributions

LJ contributed to study execution, data analysis and interpretation, manuscript preparation and final manuscript approval. SH contributed to study design and execution, data analysis and interpretation, manuscript preparation, final manuscript approval and had full access to all the data in the study and takes responsibility for the integrity of the data and the accuracy of the data analysis. TS, LLS, CF, ES, and TL, contributed to study design and execution, manuscript preparation and final approval. VW contributed to study execution, manuscript preparation and final approval. JG contributed to study design and execution and final approval of the manuscript. LMS contributed to study design and execution, manuscript preparation and final approval, data analysis and interpretation.

## Conflict of Interest

The authors declare that the research was conducted in the absence of any commercial or financial relationships that could be construed as a potential conflict of interest.

## Footnotes

^1^Ethyl pyruvate E47808–100G Sigma-Aldrich, St Louis, MO, USA

^2^PAXgene Blood RNA Tubes, Qiagen, Valencia, CA, USA

^3^Millex-GS 0.22 μm sterile millipore filter, Merck Millipore, Burlington, MA, USA

^4^Lactate Plus, Nova Biomedical Waltham, MA USA

^5^PAXgene Blood RNA kit No. 762164, Qiagen, Valencia, CA, USA

^6^RNase-Free DNase Set, Qiagen, Valencia, CA, USA

^7^High-Capacity cDNA Archive Kit, Applied Biosystems, Foster City, CA, USA

^8^7500 Fast Real-Time PCR system, Applied Biosystems, Foster City, CA, USA

^9^Taqman probes, Applied Biosystems, Foster City, CA, USA

^10^SAS Institute Inc, Cary, North Carolina, USA.

## Supplementary Material

Supplement 1 Data questionnaire completed for each horse enrolled in the study by the attending clinician at the hospital site.
