# Supplementary material for: Multicenter Placebo-Controlled Randomized Study of Ethyl Pyruvate in Horses Following Surgical Treatment for ≥ 360° Large Colon Volvulus
Source: Front Vet Sci. 2020 Apr 21;7:204. doi: 10.3389/fvets.2020.00204 (PMC7187886; doi:10.3389/fvets.2020.00204)
Supplement: Supplement 1 — Data questionnaire completed for each horse enrolled in the study by the attending clinician at the hospital site. [file Table_1.DOCX]

**Ethyl Pyruvate in Large Colon Volvulus Study #____________**

Name -_________________________________Case number____________________________

Owner’s name___________________________Phone number___________________________

Date of admission________________________Surgery date_____________________________

Breed_______________Age_____Sex_____In foal **YES NO** Days in foal____________________

Days post foaling_______________

**Admissions data**

Colic duration______________Heart rate______PCV__________TS__________L-lactate______

Horse’s body weight_______________

**Intraoperative data**

Degree of volvulus **360 720** Enterotomy performed **YES NO** Resection performed **YES NO**

Colon biopsy taken **YES NO** Biopsy site______________________________________________

Surgery time_____Episodes of MAP < 60 mmHg **YES NO** Episodes of PaO2 < 60 mmHg **Yes NO**

**Postoperative data**

Collect **1^st^** PAXgene blood tube (10 mL)_____________

LRS ± ethyl pyruvate (150 mg/kg [1 bottle in LRS]) over 60 min.

Horse returns to stall_________+6 hours_________ +12 hours_______+18 hours___________

24 hours postoperative data

Collect **2nd** PAXgene blood tube (10 mL)________________

Heart rate_________PCV__________TS________blood L-lactate____________

Fecal consistency NORMAL COW PIE DIARRHEA HEMORRAGE NO FECES DRY FECES

48 hours postoperative data

Heart rate_________PCV__________TS________blood L-lactate____________

Fecal consistency NORMAL COW PIE DIARRHEA HEMORRAGE NO FECES DRY FECES

Did the horse experience any of these events in the hospitalized postoperative period?

COLIC THROMBOPHLEBITIS INCISION INFECTION LAMINITIS SEPTIC PERITONITIS

Survived to hospital discharge YES NO

Days in hospital_____ Days on antimicrobial drugs_____
